# Monthly Summary

**Published:** 1897-08

**Authors:** 


### Monthly Summary.

ANTI-EXTRACTION.—Yearly there are extracted by the bushel teeth and roots, firmly set in the jaws, susceptible of repair and capable of performing a duty and service ten times greater than that of the best artificial substitutes. A patient with the toothache cannot take a calm, sensible view of the situation, of the irreparable injury not only through the loss of that member, but by the change of position the others will take and the unavoidable loss of the use of its opposing fellow. Out of one hundred teeth extracted, ninety-nine should not be extracted, but should be carefully and painlessly cleaned from soft decay at the margins (not over the pulp), and filled with some non-conducting cement and kept filled, imperfectly though it may be.

There are many persons, mostly ladies, who wear jewels and pearls and expensive clothing to beautify their exterior, whilst they intrust their pearls of inestimable value to the hands of the most inexperienced cheap dentists, and get his cheapest wares at his cheapest price, then go abroad and boast of the amount ecomomized in their dental bills, when one glance at their faces and one zephyr from their mouths are most convincing that their dental services were most dearly bought at the expense of their beauty and health. The worthy cheap dentist has a fruitful field, and can do an immense good, if he would confind himself to legitimate cheap dentistry without mentioning an arbitrary fee for gold work, not equal in many cases, to half of what the material used would cost.

The patient, young or old, should be told how to brush and pick and rinse his teeth, and this fact impressed on him, that teeth decay only from the outside. and for want of proper care and cleanliness. Some dentists make their living out of their patients' neglect and ignorance of the correct laws of hygiene, just as some physicians do. With our advanced knowledge of the worth of good natural teeth, and the present improved instruments and facilities for treating sensitive cavities in all positions of the teeth in a comparatively painless anner, and the numerous materials for filling such cavities, each good in its place, there is no truth in the saying that any tooth or root can not be filled. All turnkeys, forceps, and extracting screws as constructed to remove firm teeth or roots are barbarous relics of the Inquisition. For years I have extracted teeth or roots only when they were so loose they could be removed with the thumb and finger, and most heartily wish every other member of the profession would adopt that rule. They would be gratified at the conservation and restoration Nature can accomplish. There should be no artificial dentures made by the future dentist. The artificial leg- and arm-maker could increase his business with just as much justice by recommending the amputation of all rheumatic and neuralgic limbs by the aid of gas, and starting out his victims with his substitutes, which bear the same relation to the natural limbs that the best dental substitutes bear to the natural teeth.—Dr. Wm. N. Morrison, in *Western Journal.*

---

LIFE AND DEATH IN THE EMOTIONS.--Whatever can flush a face or pale it; whatever can bathe the body in hot perspiration or chill it with dampness; whatever can nerve one with strength or prostrate with weakness; whatever can cause rupture of blood vessels by pressure, or faintness from weakening circulation, must certainly have wonderful power and influence on the standard of physical condition which we call health. Instances are on record where a mother, after being very angry, has nursed her child, and it immediately went into convulsions and died, so great had been the effort of her emotions on her milk. It is not uncommon for authors to suffer with extremely cold feet while at literary work. Many speakers (as well as soldiers) are troubled with diarrhea, or with over activity of the kidneys, immediately preceding a public effort. Indeed, the variety of manifestations of this principle is almost as great as is the number of people engaged in pursuits in which the emotional nature is taxed or the emotions play an important part.

I was once an invalid and expected to die. My physicians said: "We can do nothing to help you because you know your condition so well that we can inspire no hope in you. When we sound your lungs you hear the dull thud or the light resonance and know what it means as well as we," and there never was an improvement till hope was inspired, and I am confident never could have been.

I have patients in my care to-day whose mournful state of mind over their condition is more difficult to overcome than their disease, and more prostrating in its effects on them.—C. A. Church, M. D., in *Health*.

---

IMPROVED PORCELAIN BRIDGE.—The points in this bridge are, you are able to have 22k. gold caps on the bridge, and having a bar through the porcelain to strengthen it, its brightness and cleanliness makes it a satisfactory piece of work in the mouth. The caps are first made and a piece of platinum swaged to fit on the gum and extend up on the mesial and distal surfaces of the teeth that are capped. Then a cross of platinum or pure gold is soldered about the center of the swaged piece and a platinum rod to brace it up, and it gives you something to solder the teeth to. After this, if a long piece, standards are soldered on and the body is put on. I recommend Close's body, as Downey's, compared to Close's, is like mud compared to concrete. I consider the bridge the strongest that can be made. Then you put on the gum; you can build on the inside and slope it up to where the gum commences. After this you put caps in the mouth and put porcelain piece in position, take out, invest and solder. You make a very strong and perfect joint, and, to my idea of bridge-work, it is perfection itself.—*Stom. Gazette.*

---

THE MOST barbarous method of dentistry in the world is that practised by the Kaffirs. The Kaffir dentist places his patient on the ground, and four men hold him down. Then the operator takes a piece of sharpened ivory, steel or wood, and hacks away on the gums until the tooth is extracted.—*Dominion Dental Journal.*

TUBERCULOSIS OF THE ALVEOLAR PROCESS.—Carl Zandy ("Arch. f. klin. Chir." LII., No. 1, p. 178).—While this is considered to be a rare condition the author was able to collect thirty-seven cases from the literature of the last twenty five years. He also gives the history of a patient observed at the clinic of Bonn. The teeth are of the greatest importance in the etiology of this condition. Carious teeth are the seat of entrance for tubercle bacilli. Wounds of the alveolar process, especially those caused by extraction, are of grave importance. Whether the bacilli come from a phthisical lung or from the outer world, they need no better soil for development than the alveolar cavity left after extraction, There is no seat of predilection in this disease, and any part of the alveolar process may be affected. As a rule, other parts of the buccal mucosa are involved at the same time. It is very likely that the pulmonary lesion which is found at the autopsy is secondary to the alveolar disease. Syphilis is no bar to a tubercular involvement of the alveolar process. The disease seems to develop between the ages of fifteen and fifty. Males are more frequently affected than the females. Usually the gum will swell and become loose, soft and bleed very rapidly. This will soon be followed by ulceration, with pale, sluggish granulations. Following this the teeth will get loose and fall out and the bone may become necrotic. Pain is not very marked, but salivation is very profuse and the mouth has a very foul odor. A differential diagnosis is to be made from syphilis and carcinoma. The diagnosis will be facilitated by the presence of tuberculosis of the larynx or lungs. The best therapeutic measure is through curetting and removal of all suspicious tissues. This can be followed by applications of equal parts of lactic acid and distilled water to all recurring foci.—*Amer. Med. Surg. Bull.*, August 15th, 1896.

---

AN INTERESTING CASE OF MEMBRANOUS STOMATITIS. A case of rather exceptional interest was recorded by Mr. Stanley Colyer, at a recent meeting of the students' Society of the Dental Hospital of London. The patient, a man aged about twenty, had been sent to the Western Fever Hospital, suffering from supposed diphtheria. The membrans was not symmetrical, but was confined entirely to the left side of the mouth, covering the hinder portion of the left border of the tongue, the gum around the lower molar teeth, and the tonsil. The case being unusual, careful bacteriological examinations were made, but in no instance was the Klebs-Lœffler bacillus found. After the patient had been in hospital one or two days it was discovered that he had an abscess in connection with one of his lower molars. The offending tooth was removed, and from that time the membrane began to clear up, and soon disappeared.—*Journal of Brit. Dent. Assoc.*

---

I HAVE no sympathy for the young man who says he cannot contribute a short paper, or talk to his local society meeting, because he has nothing of real interest to say. If we only opened our mouths when we had something of great value to put forth, what a very quiet world this would be—even during a presidential campaign. As a bit of mental discipline it would be an excellent idea for the young dentists to form a habit of writing up (in their moments of enforced leisure) any cases of interest that may occur in their practice, not simply with the chastened hieroglyphics of the case book, but with an eye towards a slight literary style and finish and scientific edge strength; not necessarily for publication either, but as an evidence of good faith and interest in their profession, outside the bread-and-butter aspect.—Charles McManus, D. D. S., in "International Dental Journal."

---

ADHÆSOL is a substitute for collodion, over which it has the advantage of being a better antiseptic. This preparation is a clear amber-colored liquid, with a pleasant odor, and is neither toxic nor caustic. It dries in a few seconds on the skin, and is an adherent covering to the mucous membrane. It is prepared as follows: Macerate, Resina Kopae, 350 gm.; Benzoe and Balsam Tulu aa, 30 gm., with a mixture of Ether, 1,000 gm.; Al Thymi, 20 gm. for two days, filter and add a-nopthol, 3 gm.

INCISOR OR CUSPID CROWN.—The only way I can make an open-face crown for a cuspid or incisor, that will fit perfectly, is as follows: Take, for instance a, central incisor on the right side; the left lateral and cuspid are out, and you want to make an open face crown for the central. First, get your strip of gold a little wider than the tooth is long, and a little longer than will go around the tooth. Bring it around the tooth and with a pair of flat pliers pinch and draw tigh'ly around it until it fits perfectly. Hold it tight and with another pair of pliers, of the same kind, draw or pinch the gold down at the point of the tooth. Stick a pin through the edges, remove, and solder together. Trim edges and cut out the face in the usual way. You will find that it fits perfectly and will not take five minutes to make the crown in this way. If the tooth is broken off at one corner build it up with cement.—*Ohio Dental Journal.*

---

TEETH IN RELATION TO THE EAR, NOSE AND THROAT. Gambati called attention to the importance of not neglecting the teeth in diseases general, and especially in those of the ear, nose and throat. Disease may affect the development and formation of the teeth. The reverse is also true, a carious tooth or alveolar abscess may develop symptoms that are thought to depend, by the patient, on trouble in the ear, nose and throat. The ear especially is frequently the seat of reflex disturbance that originate from the teeth, although the nose and throat are also sometimes affected in this manner.—*Laryngoscope*, March, 1897.

---

HEARING RESTORED AFTER TWENTY-FIVE YEARS.—Bennet mentions a case in his practice where a lady, 50 years of age, whe had been deaf for twenty-five years, had her hearing restored immediately after the extraction of a number of roots. Among these were the roots of the upper wisdom teeth, which were badly exostosed, and when extracted she felt as if relieved of a pressure, which she had felt for a number of years.—*Dominion Dental Journal.*

